# Effect of Instructional Manual on Knowledge and Self-Expressed Practices among Smokers Suffering from Unstable Angina Disease

**Published:** 2011-02-01

**Authors:** T Nasrabadi

**Affiliations:** 1 Department of Nursing, Islamic Azad University of Medical Sciences, Zahedan, Iran

**Keywords:** Instructional manual, Knowledge, Self-expressed practices, Smokers, Unstable angina

Dear Editor,

Since the world came into existence, humanity has striven relentlessly to be happy.[1] The prevalence of coronary artery disease (CAD) is rising rather steeply in India. According to WHO projections, there will be 100% rise in mortality from CAD in India by the year 2015 if drastic step for lifestyle optimization are not undertaken.[2] Patients with CAD should firstly understand that, to maintain a healthy body, they have to make certain meaningful lifestyle adaptations.[3]

Modification of cardiovascular risk factors can reduce the incidence of ischemic heart disease, effectively extend survival, decrease the need for interventional procedures, and improve quality of life in persons with known cardiovascular diseases.[4] Although lifestyle modification is not unexpected after hospitalization for an acute coronary syndrome (ACS), it could be anticipated that in the current era of evidence- based medicine, with early intervention and revascularization, changes in lifestyle would be relatively minor and that patients would return to regular employment soon after discharge from hospital.[5]

Tobacco in any form, chewable or non-chewable and passive smoking are equally injurious, and are major predisposing factors for a premature heart attack and sudden death. Coronary artery disease has been seen in 80 percent of smokers.[6] Public education and training may encourage people to quit smoking and cause a negative attitude towards it.[7] Quitting smoking is associated with a substantial reduction in risk of all-cause mortality among patients with CHD.[8]

From 8.10.2008 to 8.2.2009, a quasi-experimental study was conducted among 200 unstable angina patients selected by simple randomized sampling method who were admitted at Ruby Hall, Jehangir and NM Wadia Hospital in Pune city, India. The research method adopted for the study was quasiexperimental with one group pre-test and post-test design. The instrument used for data collection was the Modified Life Style Assessment Questionnaire. The collected data were organized and analyzed according to objectives of the study using distribution of demographic variables in frequency and percentage.

In demographic variables, most of the participants were in the age group 45-54 years (66%), 78% were male, 87% were married, 26.5% were university graduates and 22% were employed. Sixty four percent of those employed had 8000 Rs or more monthly incomes, 58% were couples and 37.5% had more than 5 family members. Fifty one percent had mixed dietary habits and 90% patients had a family history of heart disease.

Fifteen percent of patients smoked cigarette in the pre-test while in the post-test 10% of patients were smokers. Eighty eight percent did not smoke cigarette before the age of 20. Fifty two percent in pre-test and 67% in post-test smoked 5-10 cigarettes per day. Fifty five percent smoked cigarette for more than 15 years. The majority of smokers (40%) were less than 1 hour in the pre-test.

Knowledge about quitting of smoking [pre-test mean was 5.78, SD=0.59 and post-test mean was 5.93, SD=0.57 (t value: 2.58, p-valu: < 0.005)](Table 1). Self expressed practices about regular quitting of smoking [pre-test mean was 4.6, SD=1.0 and post-test mean was 5.9, SD=0.5 (t value: 16.44, p-value: < 0.000)]. The data suggest that patients have deficit of knowledge and self-expressed practices regarding quitting of smoking of unstable angina on pre-test. The study revealed that the instructional manual on unstable angina for patients who were smokers was effective in quitting of smoking.

**Table 1:** Comparison of pre-test and post-test score in knowledge and self-expressed practices about smoking (N=200)

**Smoking **	**Knowledge **				**Self expressed practices **			
	**Mean **	**Standard Deviation **	**‘t’ Value **	**‘p’ Value **	**Mean **	**Standard Deviation **	**‘t’ Value **	**‘p’ Value **
Pre-test	5.78	0.59	2.583	0.005	4.6	1.0	16.44	<0.001
Post-test	5.93	0.57			5.9	0.5
